# USING SMALL MOLECULES TO IDENTIFY CRITICAL HOST-CELLULAR PATHWAYS FOR BRUCELLA INFECTION

**DOI:** 10.51894/001c.123036

**Published:** 2024-08-30

**Authors:** Thomas Kim

**Affiliations:** 1 College of Osteopathic Medicine Michigan State University

## 60

### INTRODUCTION/BACKGROUND

Brucella spp., a widespread group of zoonotic intracellular pathogens, pose substantial health threats to both animals and humans worldwide. While it is well-established that nearly all Brucella species can infect and multiply within mammalian phagocytes, our understanding of the specific host factors and cellular processes that facilitate Brucella survival in the host cell remains incomplete. A critical knowledge gap lies in the identification of host cellular processes that support Brucella survival during infection.

### OBJECTIVES/HYPOTHESIS

Through pharmacologic and molecular characterization of select host-dependent processes, we aim to expand our understanding of Brucella-phagocyte interactions and uncover potential novel targets for host-directed therapeutic strategies.

### METHODS

In order to identify host-cellular processes that are critical for Brucella replication in host phagocytes, we used a small molecule screening approach. We screened compounds from FDA-approved small molecule libraries to leverage their known mechanisms of actions and thereby infer host cellular processes that may be critical for Brucella survival. In collaboration with the Michigan State University’s Assay Development & Drug Repurposing Core (ADDRC), we screened over 8000 small molecules from a library of well-characterized and uncharacterized small molecules in three complementary assays. We evaluated the effect of each drug on (a) intracellular growth of *Brucella ovis* (b) axenic growth of *B. ovis*, and (c) cytotoxic effects on THP-1 macrophages. By conducting three independent screens, we were able to filter and select for small molecule candidates that were inhibitory to Brucella in the intracellular context and not directed to the bacterium axenically.

### RESULTS

Ca2+ channel blockers, such as nicardipine, were among the top classes of small molecule candidates in our B. ovis screen, and our follow up studies revealed similar activity against the human and BSL-3 pathogen *Brucella abortus*.

### DISCUSSION/CONCLUSIONS

Host Ca2+ homeostasis has been broadly investigated in other intracellular pathogens, however its importance for Brucella infection is relatively undefined. These preliminary data show that FDA approved Ca2+ modulators are an effective host-directed small molecule in vitro and more importantly show that Ca2+ homeostasis may play an essential role in Brucella pathogenesis in the macrophage.

